# Supplementary material for: Chromothripsis during telomere crisis is independent of NHEJ, and consistent with a replicative origin
Source: Genome Res. 2019 May;29(5):737–49. doi: 10.1101/gr.240705.118 (PMC6499312; doi:10.1101/gr.240705.118)
Supplement: Supplemental Material [file supp_gr.240705.118_Supplemental_file_1.zip › contigs/annotated_contigs/DB75/contig.2.DB75_length_271_mean_cov_8.30258302583.docx]

**DB75_length_271_mean_cov_8.30258302583**

```
CATTTGATTAATGTTTGTTGAGTTCTTAAAAACATATTTTTCTCTTAACAGTACTGGCACAATTTCGGTATT|GTG|AAAAGTGGAGAT
 >chr13:63637829-63637902 + E=8e-25                                         >chr7:57606
CTGCTTTTTCTTTCTTCTTTTCATTAATCTCTATTTGGAGAAACACTGGTCTTGGAAATGAATTGAAAAGTAACATGACCTTAGACAAA
289-57606483 + E=8e-92
TCAATTAAGTTTCTTGACCCCAGACTTTTTTTTTCATCTGTATAATGCTATTTTTACTAAGGATTTATTAATGTCCTTCCAGCTCTGAT

ATTTTC
```
